# Nitric Oxide Releasing Nanomaterials for Cardiovascular Applications

**DOI:** 10.1016/j.jacbts.2023.07.017

**Published:** 2023-10-18

**Authors:** Tanveer A. Tabish, Mark J. Crabtree, Helen E. Townley, Paul G. Winyard, Craig A. Lygate

**Affiliations:** a Division of Cardiovascular Medicine, Radcliffe Department of Medicine, British Heart Foundation (BHF) Centre of Research Excellence, University of Oxford, Oxford, United Kingdom; b Department of Biochemical Sciences, School of Biosciences & Medicine, University of Surrey, Guildford, United Kingdom; c Nuffield Department of Women’s and Reproductive Health, John Radcliffe Hospital, University of Oxford, Oxford, United Kingdom; d Department of Engineering Science, University of Oxford, Oxford, United Kingdom; e University of Exeter Medical School, College of Medicine and Health, St. Luke’s Campus, University of Exeter, Exeter, United Kingdom

**Keywords:** cardiac tissue engineering, inorganic nanoparticles, nitric oxide, nitric oxide release, organic nanoparticles, stent, vascular graft

## Abstract

A central paradigm of cardiovascular homeostasis is that impaired nitric oxide (NO) bioavailability results in a wide array of cardiovascular dysfunction including incompetent endothelium-dependent vasodilatation, thrombosis, vascular inflammation, and proliferation of the intima. Over the course of more than a century, NO donating formulations such as organic nitrates and nitrites have remained a cornerstone of treatment for patients with cardiovascular diseases. These donors primarily produce NO in the circulation and are not targeted to specific (sub)cellular sites of action. However, safe, and therapeutic levels of NO require delivery of the right amount to a precise location at the right time. To achieve these aims, several recent strategies aimed at therapeutically generating or releasing NO in living systems have shown that polymeric and inorganic (silica, gold) nanoparticles and nanoscale metal-organic frameworks could either generate NO endogenously by the catalytic decomposition of endogenous NO substrates or can store and release therapeutically relevant amounts of NO gas. NO-releasing nanomaterials have been developed for vascular implants (such as stents and grafts) to target atherosclerosis, hypertension, myocardial ischemia-reperfusion injury, and cardiac tissue engineering. In this review, we discuss the advances in design and development of novel NO-releasing nanomaterials for cardiovascular therapeutics and critically examine the therapeutic potential of these nanoplatforms to modulate cellular metabolism, to regulate vascular tone, inhibit platelet aggregation, and limit proliferation of vascular smooth muscle with minimal toxic effects.

## Highlights

• NO-based therapeutics remain a cornerstone of cardiovascular pharmacology, but would benefit from targeted and controlled delivery.
• Nanomaterials can function as highly tunable platforms for drug delivery including NO via catalytic and noncatalytic approaches.
• Multiple nanomaterials have been investigated for NO delivery in vascular stents and grafts, reperfusion injury, and tissue engineering.
• Clinical translation will require multidisciplinary cooperation to optimize material properties, address safety concerns, and test in large animal models.

Despite the increasing use and significant advances made in interventional and surgical procedures, cardiovascular disease (CVD) has remained a major cause of morbidity and mortality globally for decades.^1^ The ageing population in both developed and developing countries has also led to a significant rise in the prevalence of cardiovascular diseases. The endothelium plays a crucial role in maintaining vessel homeostasis, regulating the delicately balanced processes of vascular tone and platelet activation. Vessel homeostasis is maintained by the release of various vasoactive factors from the endothelium. Vasoactive factors can be vasodilatory, including nitric oxide (NO), prostacyclin, endothelium-derived hyperpolarizing factor, or vasoconstrictive, including thromboxane A2 and endothelin-1.^2^^,^^3^ NO is one of the most important vasodilatory factors in coronary endothelium and is constitutively generated through the conversion of L-arginine and molecular oxygen to NO and L-citrulline catalyzed by the enzyme nitric oxide synthase (NOS). The essential cofactor tetrahydrobiopterin is a key regulator of cellular redox signaling and is crucial for the maintenance of vascular function, because tetrahydrobiopterin deficiency causes uncoupling of the NOS enzyme resulting in production of the superoxide anion radical (O_2_^.−^) instead of NO.^4^ The 3 distinct isoforms of NOS in mammals are neuronal NOS, inducible NOS, and endothelial nitric oxide synthase (eNOS).^5^ In the vasculature, eNOS in endothelial cells is the primary source of NO and is a crucial regulator of blood pressure, cellular proliferation, and vascular tone. NO continuously diffuses to vascular smooth muscle cells where it stimulates soluble guanylate cyclase (sGC) to produce cyclic guanosine monophosphate (cGMP), thereby activating protein kinase G, which then phosphorylates multiple target proteins resulting in smooth muscle relaxation and vasodilation.^2^^,^^5^ Neuronal NOS, the main endogenous source of myocardial NO, regulates cardiac inotropy and relaxation, and modulates intracellular Ca^2+^ homeostasis and signaling pathways including nitroso-redox balance.^6^ Hence, it is no surprise that low bioavailability of NO critically drives the progression of CVD. A full exploration of the critical roles of NO and the mechanisms that regulate NO bioavailability in cardiovascular systems are beyond the scope of this review, but these topics have been extensively reviewed elsewhere.^5^^,^^7^^,^^8^

NO down-regulates the expression of the prothrombotic protein tissue factor and inhibits the gene expression of adhesive proteins by endothelial cells, thereby exerting anti-inflammatory effects by inhibiting the adhesion of leukocytes. NO also inhibits mast cell activation and mast cell-dependent inflammatory events that contribute to destabilization of the atherosclerotic plaque.^9^^,^^10^ NO has antiproliferative effects on smooth muscle cells (SMCs) and therefore prevents neointima hyperplasia in response to injury.^11^ Thus, NO has wide-ranging vasoprotective, antiatherosclerotic, and antithrombotic roles.^11^ Drugs that may release or produce NO locally represent a significant breakthrough for the control and treatment of CVD. However, their overall success is limited by the physicochemical properties of NO, which as a diatomic gaseous molecule with one unpaired electron, is highly reactive with free radical oxygen-centered species.^12^ Thus, NO has a high diffusion rate, but a short biological half-life (in the order of 0.5-5 seconds), thereby limiting the diffusion distance to around 200 μm. NO can be rapidly oxidized to highly toxic nitrogen dioxide in the presence of molecular oxygen. Due to such limitations, there is a growing interest in developing novel formats for the controlled, predictable, and targeted release of NO. Over the last few decades, different NO delivery systems have been devised and investigated in the form of NO donors (such as organic nitrates, nitrites, thionitrites, *S*-nitrosothiols (RSNOs), *N*-diazeniumdiolates (NONOates), metal-NO complexes,^13^^,^^14^ NO-releasing nonsteroidal anti-inflammatory drugs,^15^ small molecule gas-releasing prodrugs,^16^ peptides,^17^ proteins,^18^ polymeric membranes,^19^ hydrogels,^20^ scaffolds, and nanoparticles (silica, gold, liposomes, dendrimers, and so on)^21^ to store and release NO in a controlled and therapeutically meaningful manner.

Among the previously listed approaches, nanoparticles (NPs) have garnered attention recently because of their remarkable features, which include ultra-small size, high surface area, chemical reactivity, and ease of functionalization. The storage and release of NO by NPs heavily depends on these fundamental features as well as the NO source. NPs offer several advantages over conventional donor drugs, such as the ability to control release rates and to target specific sites of action. Targeted delivery of NO-releasing NPs has been widely reviewed for cancer,^22^ antimicrobial,^23^ and medical devices^24^; however, the study of NO-releasing/generating NPs for CVD is still in its infancy. In this review, we explore the basic concept of NPs and their key characteristics for use in biomedical applications. In particular, we highlight recent developments in the field of NO-releasing/generating NPs for the targeted, controlled, and long-term release kinetics of NO along with potential future directions.

## Clinical Applications of NO Donor Drugs

To provide context, we will first briefly discuss the most clinically important NO interventions for the treatment of CVD. At the simplest level, NO is used, nonformulated, as a medical gas for the treatment of pulmonary arterial hypertension and hypoxic newborns.^25^ Administration by inhalation provides selectivity, particularly because NO is rapidly inactivated in blood, which minimizes systemic adverse effects but also limits the utility of this approach for other conditions.^8^

Low molecular weight organic nitrates, eg, glyceryl trinitrate (GTN), have been in clinical use since the 1870s, although the NO-dependent mechanism was not understood until the late 1970s.^8^ GTN releases 1 molar equivalent of NO upon activation by mitochondrial enzymes, and sublingual GTN has been extensively used to treat acute angina pectoris providing rapid relief of symptoms for a short duration. Other nitrates, such as isosorbide dinitrate and isosorbide-5-mononitrate, are slower onset, but with a prolonged duration of action making them more suitable for angina prophylaxis (eg, via oral or transdermal delivery).^26^

Nitrates are also used in the treatment of heart failure, because they cause vasodilation, thereby reducing venous return, unloading the heart and increasing stroke volume.^27^ The combined use of oral isosorbide dinitrate and hydralazine has been shown to improve cardiac function and survival in patients with chronic congestive heart failure,^28^ particularly in African-American patients, in whom NO bioavailability may be disproportionately impaired.^29^ Intravenous nitrates may also be used for the treatment of acute heart failure to alleviate pulmonary congestion. However, caution is required in conditions where excessive cardiac unloading may result in hypotension, eg, left ventricular hypertrophy and severe aortic stenosis.^27^

Continuous administration of virtually all organic nitrates results in gradual loss of efficacy because of a complex and multifaceted phenomenon termed nitrate tolerance (reviewed by Munzel et al^30^). Classically, this is observed as a rebound effect, whereby withdrawal of nitrates results in anginal symptoms that are worse than before treatment. Hence, dosing regimens for angina typically incorporate a nitrate-free period (eg, overnight) to minimize tolerance, but with the commensurate loss of 24-hour protection. Multiple mechanisms are likely to contribute to nitrate tolerance; these include impaired enzymatic drug activation resulting in reduced NO production, and nitrate resistance caused by desensitization of the sGC signaling pathway.^31^^,^^32^ Another key contributor is nitrate pseudotolerance, which arises from overcompensation by neurohormonal pathways in response to sustained vasodilation, eg, increased sympathetic stimulation, activation of the renin-angiotensin-aldosterone system, and increased sensitivity to endogenous vasoconstrictors. It is likely that an increase in oxidative stress underpins many of these mechanisms, with nitrate treatment shown to increase production of superoxide anion via nicotinamide adenine dinucleotide phosphate oxidases and mitochondria. This superoxide rapidly reacts with NO, effectively reducing NO bioavailability, but also forming peroxynitrite (ONOO^−^), which promotes NOS uncoupling and a vicious cycle of more superoxide generation. In addition, superoxide and peroxynitrite inhibit sGC in smooth muscle cells and reduce generation of the vasodilator prostacyclin.^30^^,^^33^ Hence, the administration of organic nitrates that also have antioxidant activity (eg, pentaerythritol tetranitrate) or in combination with antioxidant compounds may be particularly advantageous. Common but debilitating adverse effects such as headache and hypotension also limit the use of nitrates with severity varying dependent on pharmacokinetic profile and vascular selectivity.^30^

Other associated classes of drug augment NO signaling, eg, phosphodiesterase inhibitors, which increase cGMP levels by preventing enzymatic degradation, or Vericiguat, which stimulates soluble guanylate cyclase to restore cGMP levels. These agents may have utility in circumventing nitrate tolerance by improving NO sensitivity alongside the use of neurohormonal inhibitors to combat pseudotolerance. For example, Vericiguat is currently licensed in the United Kingdom for the treatment of heart failure with reduced ejection fraction, with the advantage over nitro-vasodilators that long-term administration does not produce tolerance.^27^ Other NO donors available in clinical settings include a lozenge for lowering blood pressure in patients with prehypertension, nitroprusside for the treatment of hypertension, and latanoprostene bunod for lowering of intraocular eye pressure in patients with glaucoma.^34^

Nitrates and nitrites are also the breakdown products of NO metabolism, but recycling pathways exist for the regeneration of NO. Nitrates in the bloodstream accumulate in saliva where oral bacteria convert it to nitrite, which in turn can be reduced to NO in blood and tissues via a variety of mechanisms.^5^

## NO-Releasing Compounds in Development

**Figure 1:**
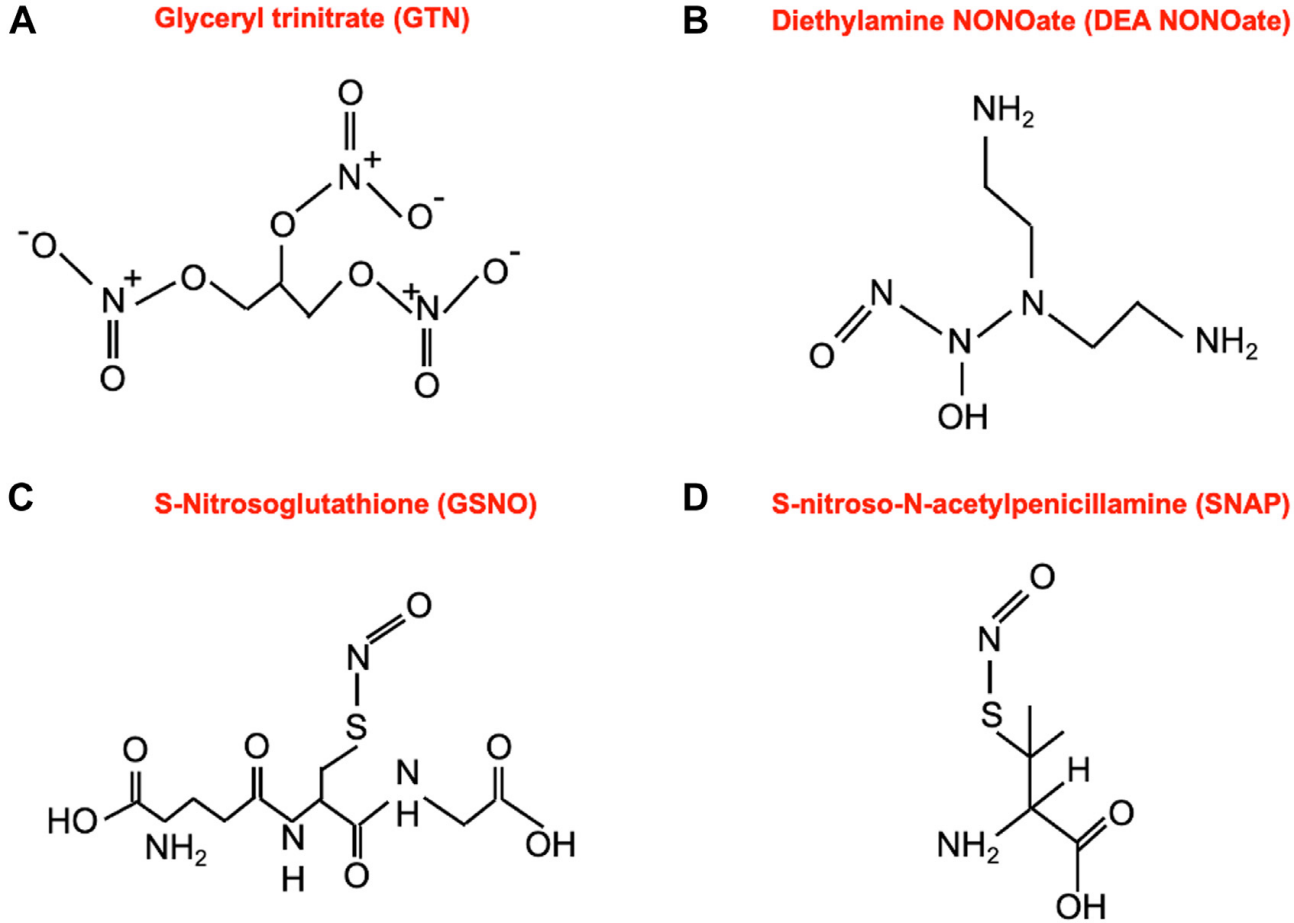
Chemical Structures of Some NO Donors (A) Glyceryl trinitrate (GTN), (B) DEA N-diazeniumdiolate (NONOate), (C) *S*-nitrosoglutathione (GSNO), (D) *S*-nitroso-*N*-acetylpenicillamine (SNAP). GSNO and SNAP are S-nitrosothiols.

Over the past few decades, NO donors and precursors, such as diazeniumdiolates (NONOates), *S*-nitrosothiols (RSNOs), and arginine, have emerged as potent enhancers of NO signaling. The chemical structures of the most commonly used NO donors are shown in Figure 1. Among these, NONOates are commonly studied because of their capability to release NO rapidly under physiological conditions (37 °C, pH 7.4). NONOates are adducts of NO dimer bound to nucleophilic residue via a nitrogen atom. When hydrolyzed, they release 2 molar equivalents of NO per mole of donor,^35^ and the release rate can be precisely controlled from seconds to days by changing the amount and degree of both hydrogen bonding and amine precursors.

RSNOs represent the addition of NO to a cysteine peptide via a S-NO bond. They can be low or high molecular weight depending on the “R” group, which will therefore greatly influence the biological properties. RSNOs can be endogenous or laboratory synthesized and act as NO donors and/or mediators of NO-signaling via post-translational modification of proteins. Important examples include *S*-nitroso-*N*-acetylpenicillamine (SNAP), *S*-nitrosoalbumin, *S*-nitrosohemoglobin, *S*-nitrosocysteine, and *S*-nitrosoglutathione (GSNO).^36^^,^^37^ RSNOs do not undergo spontaneous release of NO because of the low energy S─NO bond (≈150 kJ mol^−1^).^38^ However, NO release can be facilitated in several ways: 1) transition metal ion-mediated catalysis (eg, by Cu^+^); 2) interactions with ascorbate; and 3) photocatalysis.^39^

Despite the clinical utility of low molecular weight NO donors, they have a number of limitations, eg, their stability under biologically relevant conditions and limited NO payloads. NO release is typically uncontrolled and nonspecific, limiting the delivery of therapeutic doses to the target site. However, to address these clinical challenges, low molecular weight NO donors can be conjugated or loaded to a variety of nanoparticles for targeted, controlled, and sustained delivery of NO.

## Nanomaterials and Their Fundamental Properties

**Figure 2:**
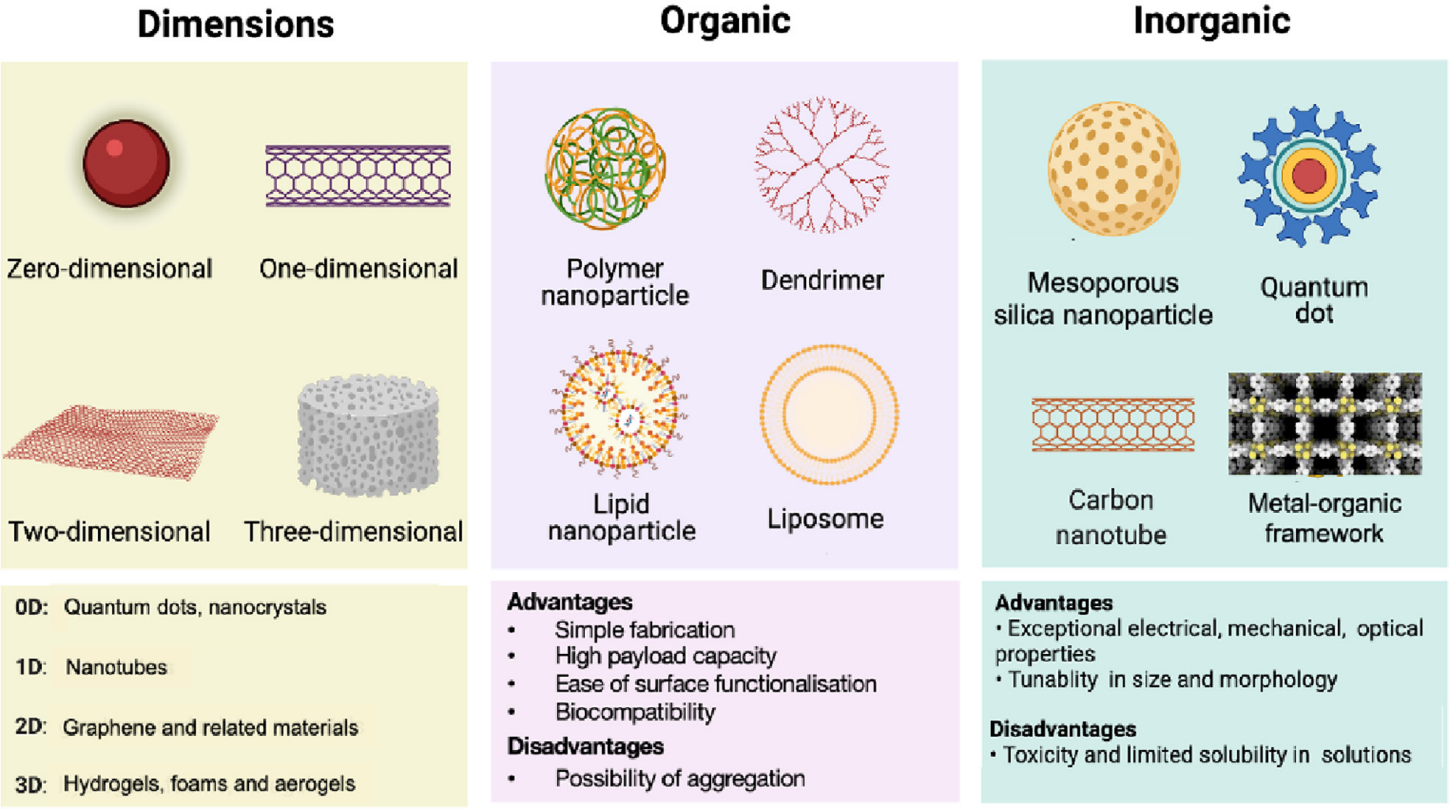
Dimensions (0D, 1D, 2D, and 3D) and Classes of Nanomaterials (Organic, Inorganic) Each class has advantages and disadvantages in terms of synthesis, functionalization, solubility, toxicity, and drug loading. D = dimension.

Nanomaterials are very small-scale chemical substances, typically 1 to 100 nm in at least 1 dimension. Nanomaterials can be classified based on their origin (natural or synthetic/engineered), composition (organic, inorganic), size, or applications (biomaterials, electronic materials, magnetic materials).^40^ NPs are further classified by dimensions, such as zero-dimensional (eg, quantum dots),^41^ 1-dimensional (eg, nanotubes, nanorods),^42^ 2-dimensional (eg, graphene, boron nitride, molybdenum disulfide), or 3-dimensional (eg, foam, aerogels, hydrogels, polymeric nanocomposites).^43^ The archetypal examples of organic NPs are polymeric, dendrimer, liposomes, and lipid NPs, whereas the archetypal examples of inorganic NPs are mesoporous silica NPs, quantum dots, carbon nanotubes, and metal-organic frameworks (MOFs) (Figure 2).

Engineered nanomaterials are designed and assembled to optimize interactions with functional payloads and to take advantage of the unique properties of their ultra-small size and high surface-to-volume ratios. These result in high chemical reactivity and quantum confinement effects,^40^ whereby the motion of electrons is restricted to specific energy levels, which significantly enhances optical, electrical, and magnetic properties.^44^^,^^45^ Thus, NPs possess physicochemical and mechanical properties that are unique and tuneable.^46^

Nanomaterials are generally prepared by 2 main wet chemical methods: the top-down and bottom-up approaches. The top-down approach involves the decomposition of larger precursors into smaller units, and then these units are converted into NPs. Typical examples of this approach are grinding,^47^ chemical vapor deposition,^48^ or physical vapor deposition.^49^ The bottom-up approach, also known as the “building-up” approach, involves the disintegration of a molecular precursor into smaller constituent parts that are then grown into colloids. Typical examples of this approach are chemical reduction,^50^ the sol-gel method,^51^ biochemical synthesis,^52^ and spinning.^53^ To date, a large number of nanomaterials have been used in a wide variety of commercial applications including sunscreens, cosmetics, sporting goods, stain-resistant clothing, tires, and electronics, as well as in medicine.

Polymeric NPs can be assembled to precisely control drug release, and they have good biocompatibility. Different drugs and biomolecules can be incorporated into the core of NPs, which are conjugated to a polymeric matrix or nanofiber. The conjugation of particles with polymers improves their drug loading efficacy for both hydrophobic and hydrophilic molecules. Such NPs can be further classified into different morphologies such as micelles or dendrimers, which are highly branched, highly symmetrical, tree-like macromolecules also referred to as hyperbranched polymers.^54^ Dendrimers have widely been reported to have high drug storage capacity and to transport the drug with minimal toxicity to normal cells.^55^ Liposomes have been one of the most widely used nanocarriers because of their multilayered morphologies that are largely adaptable to cellular environments^56^ and the layered morphologies also allow rapid cell infiltration.^57^ Excellent biocompatibility and biodegradability, as well as an inert nature, make liposomes a viable nanocarrier material without further modifications. Liposomal formulations have been reported as drug carriers for the controlled release of biomolecules and some have been approved for clinical applications, eg, liposomal amphotericin is used to reduce side-effects.

Inorganic NPs, such as silica, silver, gold, iron, titanium, carbon nanotubes, and graphene, have been developed in a wide variety of sizes and shapes and have also been utilized for drug delivery, light-mediated therapeutics (photodynamic therapy and photothermal therapy), and diagnostics (cardiac magnetic resonance, fluorescence imaging). Among the new class of nanomaterials, MOFs, comprising metal ions coordinated to organic linkers to form 1-, 2-, or 3-dimensional structures, have also received attention because of their exceptionally high specific surface area, tunable surface chemistry, and ease of functionalization with biomolecules.^58^

The ease of chemical modification on the surface of NPs facilitates the tailoring of the storage/release capacity, flux, and duration of NO. An early study on the development of NO-releasing materials was reported by Larry Keefer and his team^59^ where they used a polymeric matrix for the storage of NO (in the form of N_2_O_2_^−^) to adjust the time course of NO release. Organic NPs largely comprise polymeric NPs, micelles, liposomes, dendrimers, nanofiber composites, and hydrogels.^60^ Typical examples of NO-releasing inorganic NPs are metallic NPs (gold,^61^ silver,^62^ copper^63^), mesoporous silica,^64^ zeolites,^65^ and MOFs.^66^ An early study on the therapeutic potential of NO-releasing NPs in CVD was reported by Taite and West^67^ in 2006. They used a peptide synthesis approach in which 9-fluorenylmethoxycarbonyl (Fmoc groups) were used to protect the N-terminus, thereby allowing the formation of a branching structure of dendrimers. Dendrimers prepared in this way were used for the targeted release of NO over approximately 60 days to control vascular SMC proliferation and inhibit platelet adhesion to thrombogenic surfaces. In 2009, Nishikawa et al^68^ fabricated polysiloxane NPs (average size of 80 nm) by the reaction of sugar-lactones with amine-functionalized polysiloxane and studied localized NO release triggered by polysiloxane NPs in human aortic endothelial cells, revealing that NPs entered into cells via pinocytosis vesicles and that the cellular uptake of NPs mediated NO release, ie, that the enzymatic activity of eNOS can be triggered by targeting caveolae with NPs.

## Strategies for NO Releasing and Generating Nanomaterials

There are 2 major approaches to exploit the therapeutic release or generation of NO using NPs: catalytic and noncatalytic.^66^ Within the catalytic approach there are 4 main strategies:

1. *Enzyme prodrug systems—*In these systems, NO-releasing prodrugs are immobilized onto the surface of NPs for subsequent enzymatic activation. For example, prodrugs that are converted in vivo to spontaneous NO-releasing compounds via the action of esterases^69^ or glycosylated NONOates that are activated by endogenous β-galactosidase. To provide greater tissue specificity, glycosylated NONOates have been chemically modified so they are only cleaved by a specific mutant galactosidase. This mutant enzyme was implanted in tissues within a hydrogel, such that systemic delivery of glycosylated NONOate produced only local NO generation.^70^
2. *The attachment of transition-metal ions to NPs—*Endogenous S-nitrosothiols can spontaneously decompose to NO in a reaction that is catalyzed by Cu^2+^.^71^ Using this approach, Jiang et al^72^ reported the generation of NO from a layer-by-layer structure of copper-loaded titanium nanotubes (average diameter 30 nm).
3. *Nanozymes—*These are nanomaterial-based artificial enzymes which effectively mimic the catalytic sites of natural enzymes. They offer advantages in terms of cost-efficient synthesis, stability in biological media, and biodegradability (see specialized reviews for more on this topic^73^, ^74^, ^75^).
4. *External stimuli—*Several external stimuli, such as light, ultrasound, and x-rays, have also been used to achieve control over the NO release kinetics. The NO release profile from the donors can be changed by varying the intensity of these stimuli. There has been widespread interest in developing such external stimuli responsive NO release in cancer and infections; however, they have not been explored for CVD (see specialized reviews for more on this topic^76^^,^^77^). A few studies have reported the catalytic generation of NO by incorporating nanozymes such as titanium dioxide films,^78^ a copper-catecholic-selenocystamine framework,^79^ electrospun Cu-MOF NPs into poly(ε-caprolactone) (PCL),^80^ and PCL-based vascular graft under the catalysis of ascorbic acid.^81^

The noncatalytic approach involves chemical modification of NPs to store NO sources (eg, NO gas, NONOates, SNAP) via encapsulation into the cavities or surface, which is then released in tissues upon interaction with moisture, light, or ultrasound. For example, to achieve the controlled, slow, and sustained release of NO, studies have demonstrated the use of sequestered NO gas^82^ and of RSNOs using thiol functionalized dendrimers^83^ or gadolinium-oxide-based paramagnetic NPs.^84^ Because of their high specific surface area, nanomaterials such as MOFs have the ability to store the largest amounts of NO. This storage capacity is also dictated by the type of NO source, stability, and storage conditions. Hence, leaching of the payload is a limitation when using low molecular weight NO donors such as organic nitrates.

One unique approach has utilized magnetic NPs complexed to lentivirus for targeted gene delivery of eNOS specifically to endothelial cells (ECs). In an initial study, aortic ECs were transfected under cell culture conditions, such that they became strongly magnetic and overexpressed functional eNOS protein. These cells were then administered into the lumen of a perfused aorta where the endothelium had been injured and a specially designed array of magnets used to encourage EC recolonization in the desired radial formation on the vessel wall. In both ex vivo and in vivo experiments, improvements were observed in endothelial layer regeneration and in endothelial-dependent relaxation.^85^ Subsequent experiments eschewed the cell therapy approach for direct administration of lentiviral-magnetic NP complexes to isolated perfused vessels. Application of the magnetic field was able to enhance contact time and improve gene transfection efficiency without the need to stop flow, resulting in improved vascular function.^86^ These strategies have the potential to greatly improve targeting of gene therapy to the damaged endothelium. However, transgenic eNOS may still become uncoupled and dysfunctional if there is an insufficient supply of substrates and cofactors in the damaged vessel.

**Figure 3:**
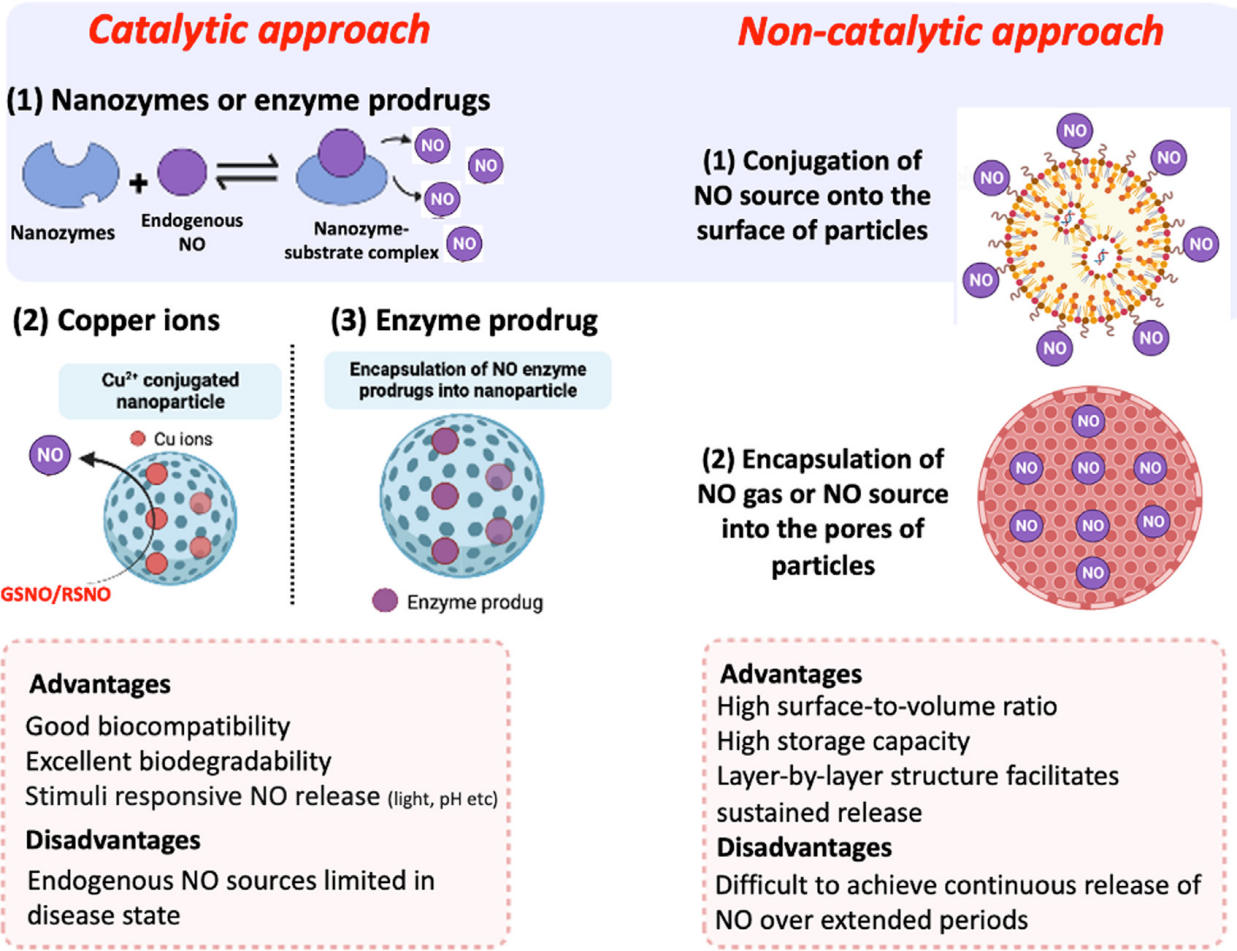
A Schematic Diagram Showing the NO Releasing and Generating Platforms Based on Catalytic and Noncatalytic Approaches NO can be generated via catalytic decomposition of natural sources of NO using nanozymes. Nanozymes are NP-based artificial enzymes that efficiently mimic the catalytic sites of naturally occurring enzymes. NO can also be delivered via incorporation of NO sources into nanoparticles.

Both catalytic and noncatalytic approaches offer advantages and limitations (schematically shown in Figure 3). For instance, low concentrations of endogenous NO substrates at diseased sites restrict the clinical use of some catalytic approaches (eg, for the prevention of restenosis). Similarly, the requirement for continuous release of NO over extended periods, such as days, weeks, and months, has not been fully demonstrated using noncatalytic approaches. Table 1 summarizes the NO release profiles of various types of nanoformulations for use in different cardiovascular disease models.

**Table 1:** Examples of NO-Releasing NPs for Cardiovascular Applications

Application	Source of NO	Key Design Features	NO Release Profile Measured in PBS	Model System	Outcomes
Stent^140^	NO gas	Assembly of nanofibers (7-8 nm diameter) coating a 316 L stainless steel stent	4.8 μmol NO over 30 days	In vitro: HUVECs and aortic SMCs	Increased proliferation of endothelial cells and inhibited the proliferation of SMCs
Stent^78^^,^^141^^,^^142^	Various coatings with catalytic decomposition of endogenous RSNO	TiO_2_ film Metal-catecholamine assembly Copper-dopamine (Cu^II^-DA) network Immobilization of selenocystamine on nanomaterials followed by coating on a 316 stainless steel stent	TiO_2_ film: Generation of NO with a rate of 1 × 10^−10^ mol cm^−2^ min^−1^ Metal-catecholamine assembly: over 30 days ranging 0.5-4 × 10^−10^ mol cm^−2^ min^−1^ Cu^II^-DA network: NO release rate: 50 × 10^−10^ mol cm^−2^ min^−1^	In vitro: smooth muscle cells In vivo: adult dog restenosis model and rabbit arteriovenous shunt model.	In vitro: NO generation in vitro inhibited platelet activation and aggregation In vivo: selenocystamine immobilized stents are endothelialized and showed significant antiproliferation properties^70^ Promoted re-endothelialization and improved antirestenosis^107^^,^^109^
Stent^99^	DETA NONOate	Liposomes Size: ≈120 and 20 nm Layer-by-layer coating on a stent	50%^a^ of NO released over 16 h	In vitro: HUVEC In vivo: castrated male pig coronary injury model.	In vitro: Increased proliferation of endothelial cells and inhibited the proliferation of SMCs In vivo: NO stent induced higher endothelial coverage of 94% ± 4% vs 34% ± 3% for bare-metal stent
Stent^143^	SNAP	SeCA/Dopa coating onto 316 L stainless steel stent. Coating thickness: 9.1-16.1 nm	0.5-4 × 10^−10^ mol cm^−2^ min^−1^ over 60 days	In vitro: HUASMC and HUVECs In vivo: New Zealand white rabbit arteriovenous shunt model Ex vivo: New Zealand white rabbit circulation thrombogenicity model	In vitro: Increased proliferation of endothelial cells and inhibited the proliferation of SMCs via up-regulation of cGMP synthesis In vivo: release of NO induced by coated stents enhanced re-endothelialization and reducing in-stent restenosis
Stent^69^^,^^79^^,^^128^^,^^144^	Various coatings utilizing GSNO	Titanium dioxide nanotube Hydrogels (composed of alginate and gelatin). Nanoscale copper-based MOF Polymeric NPs: PLGA, PEG, and PCL coated onto the surface of stainless-steel stent	Ti_2_O nanotubes: NO release rate: ∼1.5 × 10^–10^ mol cm^–2^ min^–1^. Hydrogels: NO release rate: 6.2 × 10^−10^ mol cm^−2^ min^−1^ MOF: 2.4 × 10^−10^ mol cm^−2^ min^−1^ Polymeric NPs: NO generation up to 61 ± 10% from GSNO	In vitro: HUVECs, HUASMCs, and Mouse macrophage lineage cells In vivo: rats and rabbits arteriovenous shunt model; male Bama miniature pig of arteriovenous shunt model	In vitro: coated surface significantly improved endothelial cell growth and inhibited SMC proliferation In vivo: modified stents have rapid re-endothelialization, anti-inflammation, and anti-intimal hyperplasia abilities, compared with the unmodified stents
Artificial blood vessel^80^	NO-generating coatings, Source: GSNO	Cu-MOF nanoparticles into PCL fibers Size: 438 nm		In vitro: HUVECs Ex vivo: male Sprague Dawley rats of abdominal artery replacement model	Embedded Cu-MOFs for slow release of copper ions and sustained NO production. Significantly increased endothelial cell growth while largely inhibited SMC proliferation
Vascular graft^81^^,^^138^^,^^145^^,^^146^	Catalytic decomposition of GSNO and S-nitrosated keratin	PCL based nanofiber assembly Diameter: 263 ± 90 nm POSS-PCU NO-eluting polymer-based small diameter bypass graft	NO release rate: 250 μg/mL, ∼1.4 mmol/L at 24 h	In vitro: HUVECs and HASMCs In vivo: rabbit carotid artery replacement; mouse model of atherosclerosis	Modified graft significantly increased endothelial cell growth while largely inhibited SMCs proliferation both in vitro^73^^,^^101^^,^^110^^,^^111^ and in vivo models^73^^,^^101^
Vascular injury^82^	NO gas	Liposomes of phospholipids and cholesterol	Release: 1.6 μmol over 60 min from NO/argon-containing liposomes. NO release was measured in PBS	In vitro: VSMC In vivo: male New Zealand White rabbits fed an atherogenic diet 2 wks before balloon injury of carotid artery	Liposomes taken up by SMC and significantly inhibited proliferation In vivo: NPs incubated at time of injury reduced neointimal hyperplasia and reduced arterial wall thickening 14 d postinjury
Blood vessel^147^	NO gas	PEG–Lys_5_–NO hydrogels	PEG–Lys_5_–NO hydrogels released 89% of NO over 60 d	In vitro: bovine aortic endothelial cells and rat aortic smooth muscle cells In vivo: rat carotid balloon injury model	In vitro: promoted endothelial cell growth and inhibited smooth muscle cell proliferation In vivo: NO-releasing hydrogels were applied to the outer surfaces of carotid. NO was allowed to diffuse into the vessel and intimal thickening was reduced by ∼90%
Hypoxia/reoxygenation model of cardiomyocyte injury^148^	DETA NONOate	Nanofibers	NO release: 1.26 μmol·mg^−1^	In vitro: H9c2 cells (immortalized embryonic rat heart myoblasts)	NO-releasing nanofibers prior to hypoxia induction were cytoprotective against reoxygenation injury. Inhibited the generation of hydrogen peroxide, a major contributor to oxidative damage
Myocardial ischemia/reperfusion injury^83^	SNAP + GSH	Nanometer scale 4- polyamidoamine dendrimers	NO release rate: 1,429 ppb NO mg^−1^ of SNAP s^−1^ (191 pmol NO mg^−1^ s^−1^ after 45 min)	In vitro: HUVEC and pulmonary artery endothelial cells (CPA-47 cells-calf pulmonary artery 47) Ex vivo: perfused heart of male Sprague–Dawley rats	Dendrimers localized near the cell surface and exposed to levels of GSH sufficient to initiate NO release resulting in high local concentrations of NO. Increased cell survival and reduced myocardial injury
Cardiac tissue engineering^117^^,^^124^^,^^125^	Catalytic decomposition of GSNO	Electrospun PCL/PK based nanofibrous mats PCL/keratin/Gold NP mats	10 μmol up to 36 h	In vitro: HUVECs and HUASMCs	The biocomposite selectively enhanced adhesion, migration, and growth of ECs while suppressing proliferation of SMCs in the presence of glutathione (GSH) and GSNO
Atherosclerotic plaque^138^	SNO-phospholipid	High-density lipoprotein (HDL-like) NPs Size: 13.1 ± 0.7 nm	NO release rate: 80%^a^ at 24 h	In vitro: AoSMCs In vivo: ApoE knockout fed high-fat diet for 18 weeks. NPs given 3× per wk IV for final 6 wks	In vitro: NO-releasing NPs reduced SMC migration In vivo: atherosclerotic area was 42% lower in NP-treated mice compared with controls, thereby demonstrating reduction in plaque burden

DETA NONOates = diethylenetriamine *N*-diazeniumdiolates; GSH = glutathione; GSNO = *S*-nitrosoglutathione; HASMC = human aortic smooth muscle cell; HUASMC = human umbilical artery smooth muscle cell; HUVEC = human umbilical vein endothelial cell; MOF = metal organic framework; NO = nitric oxide; NP = nanoparticle; PCL = poly(ε-caprolactone); PCL/PK = poly(ε-caprolactone)/phosphobetainized keratin; POSS-PCU; polyhedral oligomeric silsesquioxane poly(carbonate-urea)urethane; RSNO = *S*-nitrosothiol; SMC = smooth muscle cell; SNAP = *S*-nitroso-*N*-acetylpenicillamine; VSMC = vascular smooth muscle cell.

a The release data provided was insufficient and could not be used to calculate moles.

## Application of NO-releasing and -Generating Nanoplatforms for Cardiovascular Therapeutics

### Cardiovascular stents

In the cardiovascular field, stents are the most widely investigated application for NO-releasing NPs. Stents are inserted following angioplasty and conventionally consist of a metal expandable tubular mesh, which acts as a scaffold to hold open the vessel and thereby maintain blood flow.^87^ Although a substantial improvement on balloon angioplasty alone, bare-metal stents may themselves induce vascular damage and inflammation leading to thrombosis, intimal hyperplasia (characterized by SMC proliferation and collagen deposition), and renarrowing of the blood vessel, a phenomenon known as in-stent restenosis.^88^ Restenosis is most common within 12 months of stent implantation when it affects up to 20% of patients.^89^ In the last 2 decades this has been reduced to ∼2% by the use of drug-eluting stents, eg, paclitaxel and sirolimus analogues,^90^ which suppress local immune responses and are potent inhibitors of SMC proliferation. These drugs are incorporated into a polymer coating between 4 and 22 μm thick,^89^ from which they are eluted at a controlled rate. However, issues remain with drug-eluting stents, and the cumulative risk of restenosis creeps up after the first year because of neoatherosclerosis, which is driven by a number of factors, such as chronic inflammation and suboptimal endothelialization, but also by hypersensitivity reactions to polymer components and flow disturbances caused by the stent itself, leading to platelet activation and stent thrombosis.^91^^,^^92^ Given that millions of patients worldwide receive a stent annually, even a 1% to 2% restenosis rate is a sizeable problem, with 10% to 20% of these patients going on to develop recurrent restenosis.^89^

It should be noted that the use of drugs to inhibit SMC proliferation may also inhibit endothelial cell adhesion and proliferation.^92^ Therefore, current approaches can be viewed as a double-edged sword. Timely endothelialization of the vessel wall and the stent surface is important because a healthy, confluent endothelial layer produces NO to reduce vascular permeability, inflammatory cell activation, platelet aggregation, and SMC hyperplasia.^93^^,^^94^ Multiple preclinical studies have shown that exogenous NO promotes EC proliferation and migration, both in cell culture and in response to vascular injury.^95^ In part, this reflects an effect of NO to promote EC survival by preventing apoptosis, via both cGMP-mediated mechanisms to modulate antiapoptotic protein kinases and by S-nitrosation of caspases to prevent activation.^96^ Hence, an ideal treatment would *enhance* healing and rapid endothelialization as well as *reduce SMC hyperproliferation* and thrombogenesis, which makes the use of stent coatings that release or generate NO an attractive proposition.

Stents are typically fabricated by laser cutting stainless steel, titanium-nickel, tantalum, or platinum–iridium alloys.^92^ Nanomaterial-based coatings have been devised to release or generate NO in a controlled manner. In particular, layer-by-layer coating using polymers, polymeric NPs, and other classes of NPs have the potential to extend the NO release profile from hours to days and weeks.^60^ Modifying the surfaces of cardiovascular implants to release NO depends on the mechanical properties of the materials, such as stiffness, hardness, mechanical strength, viscoelasticity, shape memory behavior, and surface chemistry, which in turn determine the biological fate of such materials.^97^ The Seifalian laboratory has pioneered the use of NO-releasing NPs for stents and vascular grafts,^98^ demonstrating polymeric NPs, nanofibers, and hydrogels for the storage of SNAP and GSNO and the controlled release of NO. The results of these preclinical studies are discussed in the following text and in the next section.

Elnaggar et al^99^ used a method based on layer-by-layer deposition of liposomes (of sizes ≈120 and 20 nm) for the sustained release of NO from a modified stent. They coated stents with liposomes (the thickness of the layer was higher than ≈10 nm) encapsulating NONOate, which was trapped between layers of poly-l-lysine and hyaluronic acid–dopamine, providing a NO release profile of up to 5 days. In vitro results using human umbilical vein endothelial cells (HUVECs) exhibited significantly more endothelial cell proliferation and distinctly inhibited SMC proliferation. Scanning electron microscopy (SEM) analysis of on-stent endothelial layer formation demonstrated the effects of NO on rapid reendothelialization. Studies in pigs demonstrated efficacy in promoting arterial healing and preventing neointimal thickening.

**Figure 4:**
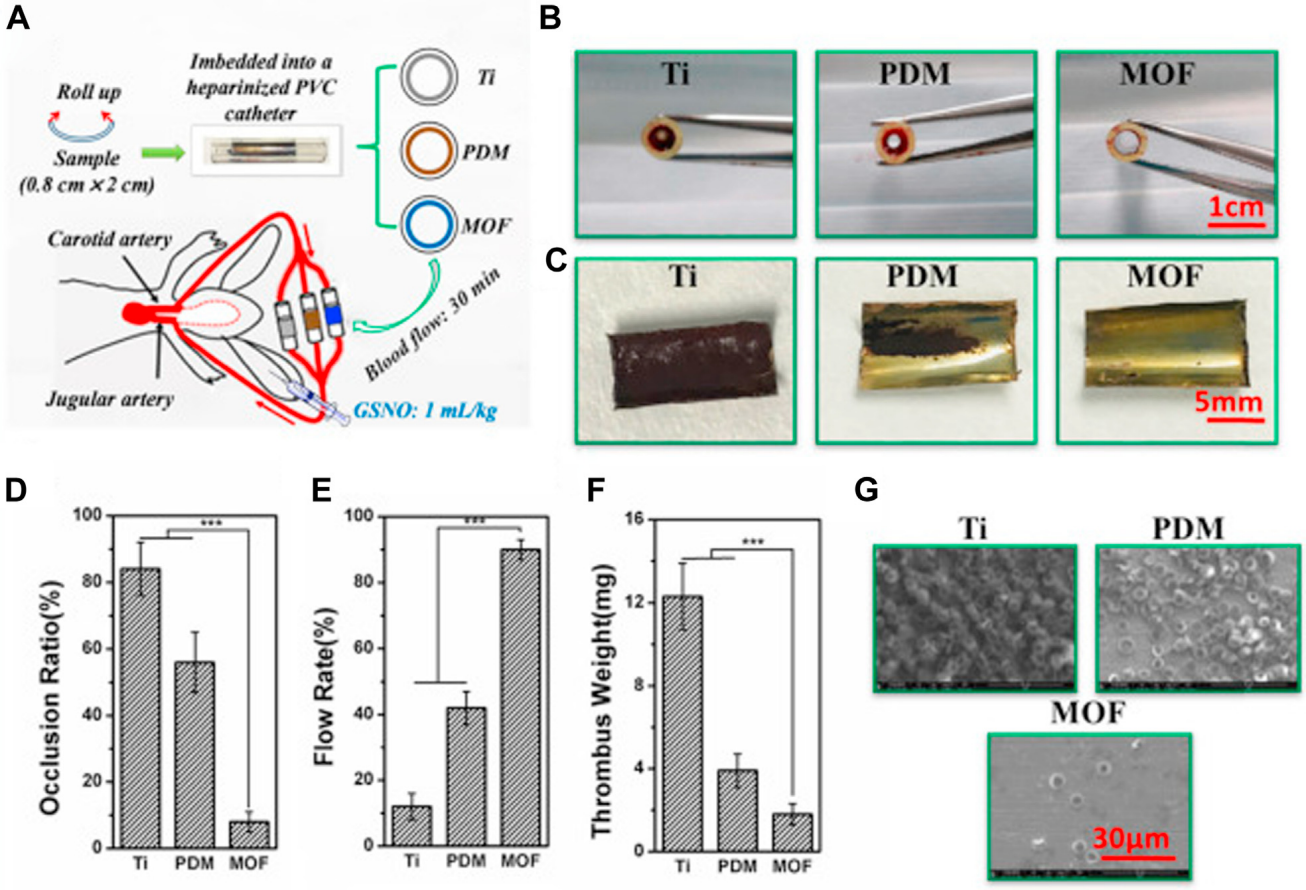
Ex Vivo Assessment of the Antithrombogenic Characteristics of the Nanoscale Cu-MOF Coating in an Arteriovenous Shunt Model (a) Schematic representation of the strategy. The scheme represents the rolling of uncoated titanium (Ti) foil and nano metal-organic framework (MOF)–immobilized Ti foil into the heparinized polyvinyl chloride (PVC) catheter, which was then connected in parallel by sterile surgical aspirators and assembled with indwelling needles. (b) Images of all the groups comprising of catheters after 30 min circulation. (c) Images of the thrombi on all the groups. (d) Occlusion ratio of a catheter by analyzing the cross-section diameter of the tube. (e) Relative blood flow kinetics at the end of the circulation in comparison to that of the initial blood flow. (f) Quantitative investigation of the thrombus formation on the surface. (g) SEM images of the thrombi. Data are presented as the mean ± SD (n ≥ 4). Reproduced with permission from Fan et al.^100^ PDM = polydopamine-coated matrix.

Fan et al^100^ reported the immobilization of nanoscale copper-based MOF (size range 10-500 nm) onto the surface of a titanium stent using polydopamine as a coating matrix, thereby allowing the copper-catalyzed generation of NO from endogenous sources. Cu-MOF were prepared by conversion of Cu(OH)_2_ to CuBTC (BTC—1,3,5-benzenetricarboxylic acid) through an acid base reaction. Copper-catalyzed generation of NO from the surface of modified 316 L stainless stent resulted in suppression of platelet aggregation via NO-cGMP signaling and considerably reduced thrombosis in an *ex vivo* extracorporeal circulation model (Figure 4), as well as reducing neointimal hyperplasia in an arteriovenous shunt model. However, this approach relies on a plentiful supply of endogenous RSNO around the implanted stent, and further optimization is required to generate therapeutically relevant amounts of NO at the site of action. The efficacy of in vivo endothelialization of this copper-based MOF coating was demonstrated by the formation of an endothelial layer on the stent (confirmed by SEM) in comparison to the attachment of red blood cells and platelets on the uncoated stent.

In a similar approach, a copper-catecholamine-selenocystamine framework crosslinked with 3,4-dihydroxy-L-phenylalanine was coated onto the surface of a stainless-steel stent through a dip-coating method by immersing the framework and stent into the reaction solution (dissolving Dopa hydrochloride, selenocystamine hydrochloride, and CuCl_2_·2H_2_O in Tris buffer) at room temperature for 24 h. This framework facilitates the interface with glutathione peroxidase (GPx) to promote catalytic activity, thereby resulting in controlled and slow release of NO from the coating.^79^ This conferred good antithrombogenic activity, improved HUVEC migration, and inhibited proliferation of human umbilical artery smooth muscle cells in vitro, as well as improved antithrombogenicity, anti-restenosis, and endothelialization in rats in vivo. The formation of an endothelial layer on the stent surface was confirmed morphologically by SEM. Thus, effective suppression of restenosis and promotion of reendothelialization can be achieved by the continuous generation of NO in a controlled manner with a copper-catecholamine framework coated stent.

Microscale polymeric materials or fibers that release or generate NO have also been explored for stent applications. Examples include poly(lactic-*co*-glycolic acid) (PLGA),^101^ poly(vinyl alcohol) and poly(vinyl pyrrolidone),^102^ thermoplastic polyurethanes,^103^ poly(methyl methacrylate),^104^ and metallic thin films including titanium dioxide,^78^ and readers are referred to specialized reviews on this topic.^98^^,^^105^^,^^106^ Such polymeric materials have shown promising results on re-endothelialization, but have limitations because of the accumulation of acidic degradation by-products, which alter pH and NO release kinetics. The NP approach advocated in this review provides additional benefits in terms of tunable properties, ease of functionalization, and high specific surface area to carry large quantities of NO. It is notable that the release rate of NO from NP-based stent coatings was similar to that of endothelial cells, ie, in the range of 0.5 to 4.0 × 10^−10^ mol cm^−2^ min^−1^.^100^

Despite promising results using NO-producing stents in vitro and in animal models, the clinical translation of this approach presents a number of challenges and issues for further study. For example, efficacy may be limited by uneven distribution of catalytic complexes, limited NO source reservoir or NO-release kinetics, coating stability, biocompatibility, and, in particular, the potential formation of toxic nitrosamines and peroxynitrite during degradation.^98^ Formulations must also be stable during storage and robust enough for percutaneous application even in difficult to reach vessels.

### Vascular grafts

The most frequently used synthetic polymer for vascular grafts is PCL due to its biocompatibility and advantageous mechanical properties. However, the hydrophobicity of PCL may induce thrombosis and intimal hyperplasia, for which the protective effect of NO has been investigated.^107^

Seifalian et al^98^^,^^107^^,^^108^ have developed a class of polymeric nanocomposites, such as polyhedral oligomeric silsesquioxane poly(carbonate-urea)urethane (POSS-PCU), to modify a variety of surgical implants, aimed at releasing NO in a targeted manner. de Mel et al^108^ reported the incorporation of SNAP and GSNO into POSS-PCU–based bypass grafts for the in situ release of NO in the presence of pulsatile flow. This study revealed that NO release from POSS-PCU helped inhibit platelet and SMC adhesion while stimulating endothelial cell adhesion tested in endothelial progenitor stem cells and SMCs. The release profile reported in this study was 10 nmol/L NO over the first 10 minutes with a continuous diminution up to day 7. Clearly, a longer duration of NO release from vascular grafts would be desirable. Zhan et al^80^ investigated the potential of electrospun Cu-MOF NPs (average size 438 nm) into PCL. MOF NPs were prepared by the reaction between organic molecules and metal ions, and vascular scaffolds were prepared by electrospinning PCL and MOF NP solution. This approach helped avoid the interaction of Cu-MOFs with serum and considerably slowed the leaching of copper ions, thereby allowing for longer-term NO catalytic capacity in serum over 6 hours. This composite promoted accelerated endothelial cell migration and supported the monolayer development of endothelial cells in HUVECs and human SMCs as well as rat abdominal artery replacement and arteriovenous shunt models. The formation and coverage of a confluent layer of endothelial cells on grafts were assessed by SEM, demonstrating a much higher endothelialization rate compared with the control graft.

### Myocardial ischemia-reperfusion injury and myocardial infarction

Following a myocardial infarction, timely reperfusion of the occluded coronary artery is the most effective intervention to minimize ischemic injury. However, reperfusion itself also induces injury because of a variety of cellular stresses, such as high intracellular calcium, rising pH, and generation of ROS, that culminate with mitochondrial depolarization and cardiomyocyte cell death. Such damage is termed ischemia/reperfusion (I/R) injury, and multiple lines of evidence suggest a protective effect of NO.^109^^,^^110^

For example, canonical NO signaling via eNOS and protein kinase G helps mediate ischemic preconditioning, whereby brief periods of ischemia before an infarct reduce subsequent myocardial injury.^111^ NO generation via eNOS is also integral to the RISK (Reperfusion Injury Salvage Kinase) pathway, which when stimulated during early reperfusion, ultimately inhibits mitochondrial depolarization and limits cell death.^112^ NO is also thought to have direct cardioprotective effects via S-nitrosation of thiols in key mitochondrial proteins, in particular, complex I. Rapid reactivation of complex I early during reperfusion is a major source of H_2_O_2_, which contributes to oxidative damage and cell death; however, reversible S-nitrosation mitigates against this by maintaining complex I in a low activity state.^113^ This protective effect has been elegantly demonstrated by the Murphy laboratory, which has created MitoSNO by covalently linking SNAP to a triphenylphosphonium cation group, providing mitochondrial targeting because of its lipophilicity and positive charge. When MitoSNO was given to Langendorff-perfused mouse hearts at the point of reperfusion, it significantly reduced infarct size and improved functional recovery.^114^ This provides proof-of-concept for the mitochondrial targeting of NO during early reperfusion, although, as with all potential I/R therapies, it is inherently difficult to target the site of injury at a time when blood flow is impaired.

Although the previous example is a small molecule approach, there is potential to target these pathways using NPs. Schoenfisch laboratory has developed and patented wide-ranging innovative NPs (porous silica, dendrimers, and other polymeric NPs) for the storage and release of NO, which have primarily been tested for the treatment of bacterial infections.^23^ In 2010, they explored the ability of functionalized dendrimer NPs for reduction of I/R injury in the isolated, perfused rat heart.^83^ Dendrimers were prepared by following a thiol-yne chemistry method. Each multibranched dendrimer was designed to optimize cellular uptake and bioavailability and was conjugated to 64 molecules of SNAP. Hearts were also exposed to varying concentrations of reduced glutathione (GSH), which represents an important variable in vivo, because it can chemically reduce S-nitrosothiols (such as SNAP) through direct transnitrosation.^115^ Thus, NO release was found to be much higher in the presence of GSH compared with dendrimer-SNAP alone, and the GSH levels effectively determined NO release kinetics.^83^ Optimal doses of dendrimer-SNAP and GSH were determined to establish a proof-of-principle reduction in infarct size, although low experimental numbers urge caution with interpretation of these findings. Further work is needed to determine the cellular uptake and fate of these dendrimers and for confirmation in clinically-relevant in vivo models.

It should be noted that NO may represent a double-edged sword in I/R injury. Cardioprotection depends on the timely and temporary delivery of controlled amounts of NO to the mitochondria. However, the coexistence of excess superoxide anion radicals can lead to formation of highly toxic peroxynitrite.^116^ Peroxynitrite is a powerful oxidizing and nitrating agent that can target multiple substrates resulting in membrane lipid peroxidation, mitochondrial damage, disturbances in cell signaling, apoptosis, and necrosis. Therefore, the release of NO from NPs using exogeneous NO sources will need to be fine-tuned to achieve optimal therapeutic effects for I/R injury.

### Tissue engineering

Cardiovascular tissue engineering holds promise for the repair and regeneration of injured myocardium, heart valves, and blood vessels, but this requires strong coupling with surrounding native tissues. In particular, vascular grafts can be problematic for small diameter vessels because of issues of patency, so there is interest in developing scaffolds to mimic the extracellular matrix that can either be seeded with stem cells in vitro or that promote natural angiogenesis to grow new vessels in situ. Challenges include biocompatibility, promoting endothelialization while suppressing SMC hyperplasia, and preventing hypercoagulation.^117^ Hence, it is no surprise that NO-generating/releasing scaffolds have been developed for vascular tissue engineering. Scaffolds are 3-dimensional porous networks formed from natural or synthetic materials, which provide an ideal 3-dimensional microenvironment to promote cell–cell communication, cell attachment, migration, differentiation, and proliferation. Such scaffolds for cardiac tissue engineering applications generally comprise of natural or synthetic materials, including polymers, collagens, silk, alginate, chitosan, and hydrogels, because of their good mechanical strength and favorable degradation profiles.^118^ Scaffolds have been used extensively for cardiac tissue engineering applications, specifically in ischemic heart diseases, and cardiac patches,^119^, ^120^, ^121^, ^122^, ^123^ but there are limited studies using NO-based technology.

The group of Shen and colleagues has published a series of papers^117^^,^^124^, ^125^, ^126^ using an electrospun scaffold of PCL/keratin mats (size 457 ± 53 nm) functionalized with gold NPs (size 22.1 ± 4.5 nm)^117^ for use as tissue-engineered vascular grafts. This represents a typical application for cardiovascular tissue engineering where vascular or stem cells are seeded onto biodegradable scaffolds to regenerate vascular tissues instead of an autologous blood vessel.^127^ In recent years, keratin, a natural component of human hair, skin, and nails, has been appreciated as a biomaterial providing excellent biocompatibility and biodegradability. Moreover, cysteine is a key component of keratin, which further facilitates the decomposition of endogenous sources of NO, catalyzed by the gold NPs. These functionalized scaffolds were able to catalyze NO generation when tested in vitro, improving HUVEC growth and inhibiting human umbilical artery smooth muscle cells viability.^117^ Further work is required to realize the potential for use in tissue engineering by testing the effects on endothelialization and SMC proliferation in vivo. There are a few studies where NO-releasing/generating scaffolds based on hydrogels^128^ or other polymers, PLGA, or PCL^129^ have been studied for use in cardiovascular applications specifically in tissue-engineered vascular grafts and have been discussed elsewhere.^130^^,^^131^ In NO-releasing tissue engineering approaches, micron-sized materials have been used as NO carriers because of their ability to store high amount of NO.^117^^,^^124^^,^^131^

### Other applications of NO-releasing NPs

Angiogenesis is characterized by the growth of new blood vessels sprouting from other pre-existing small vessels and plays a critical role in the response to tissue ischemia and wound healing as well as being a major goal in tissue engineering. Angiogenesis is promoted by several growth factors, such as vascular endothelial growth factor and basic fibroblast growth factor, which stimulate quiescent endothelial cells into a highly proliferative state.^132^ NO is an important regulator of endothelial function affecting angiogenesis.^133^^,^^134^ Yang et al^135^ developed NONOate-incorporated methoxy poly(ethylene glycol)-b-poly(lactic-co-glycolic acid) (mPEG-PLGA) NPs of 200 nm size with NO release of ∼80% in 24 hours. The induction of angiogenesis was evaluated by tube formation, which shows the formation of capillary-like structures. They demonstrated that the tubular formation increased 190% in NO-releasing NP-treated groups in comparison to the control group. The induction of angiogenesis was evaluated ex vivo using rat aorta treated with NO-releasing NPs and revealed sprouting angiogenesis. In another study, Lee et al^136^ reported DETA NONOate incorporated mPEG-PLGH-thiobenzamide NPs of 140 nm size and showed that NO-releasing NPs released up to 20 nmol over 72 hours and exhibited enhanced angiogenesis compared with the control groups in both in vitro (HUVECs and 3T3-L1) and *ex vivo* (rat aorta) models.

Endothelial dysfunction associated with a deficiency of NO is broadly acknowledged as an integral first step in the development of atherosclerosis.^137^ Hence, several groups have experimented with the systemic administration of NP NO donors. Rink et al^138^ described the synthesis of RSNO phospholipid (S-nitrosylated 1,2-dipalmitoyl-*sn*-glycero-3-phosphonitrosothioethanol) within an outer phospholipid shell composed of a naturally abundant high-density lipoprotein (HDL). HDL NPs were synthesized by reductive methylation of apolipoprotein A-I by 3H-formaldehyde. Such NO-releasing HDL NPs retain many of the properties of natural HDLs, thereby improving biocompatibility and targeting to the vasculature. ApoE knockout mice were fed a high-fat diet for 18 weeks, with treatment given by intravenous injection 3 times/wk from week 12 onwards. Compared with vehicle-administered control subjects, treatment with functionalized HDL NPs reduced atherosclerotic plaque burden by 42%.^138^

Mohamed et al^139^ developed NO-releasing polymeric NPs based on a polyvinylpyrrolidone composite incorporating nitrite and a reducing agent as the NO source. Polymeric NPs were prepared via ionotropic gelation technique. In aqueous solution, steady-state NO release was obtained within 7 minutes and lasted for 2 hours, followed by a 50% lower release phase that was sustained for at least 8 hours. NO-releasing NPs caused dose-dependent relaxations of pulmonary arteries taken from mice with hypoxia-induced pulmonary artery hypertension without altering the viability of endothelial cells.

Huang et al^82^ formulated liposomes comprising phospholipids and cholesterol that encapsulate NO gas. Liposomes were prepared by modified pressured-freeze method. NO-release kinetics could be controlled via the incorporation of argon, with formulations releasing 1.6 μmol over 60 minutes and providing sustained and slow release over 8 hours. Delivery of NO to vascular smooth muscle cells was 7-fold higher using the liposomes compared with uncaged NO, and remained high even in the presence of hemoglobin, indicating that this formulation improves bioavailability by protecting against NO scavenging. Efficacy was tested in a rabbit model of carotid artery vascular balloon injury, where NO-releasing liposomes were incubated in the artery for 2 minutes immediately after injury, and the damage was assessed by histology 2 weeks later. NO-releasing liposomes significantly reduced neointimal hyperplasia, and there was a 41% reduction in arterial wall thickening.^82^ Table 1 summarizes NO-releasing NPs for use in cardiovascular applications.

## Translational Considerations and Future Outlook

This review has examined a wide range of NO-releasing and -generating NP formulations designed and tested for the potential treatment of CVD. Promising proof-of-principle data have been presented for a variety of different applications, such as vascular stents and bypass grafts, I/R injury, atherosclerosis, and tissue engineering (Central Illustration). There are currently 214 NO-related clinical trials for CVD applications registered (visited on July 24, 2022) at ClinicalTrials.gov. However, there are currently no clinical trials on NO-releasing NPs for CVD applications, and this reflects the need for additional preclinical studies to overcome a number of limitations and unknowns.

**Central Illustration:**
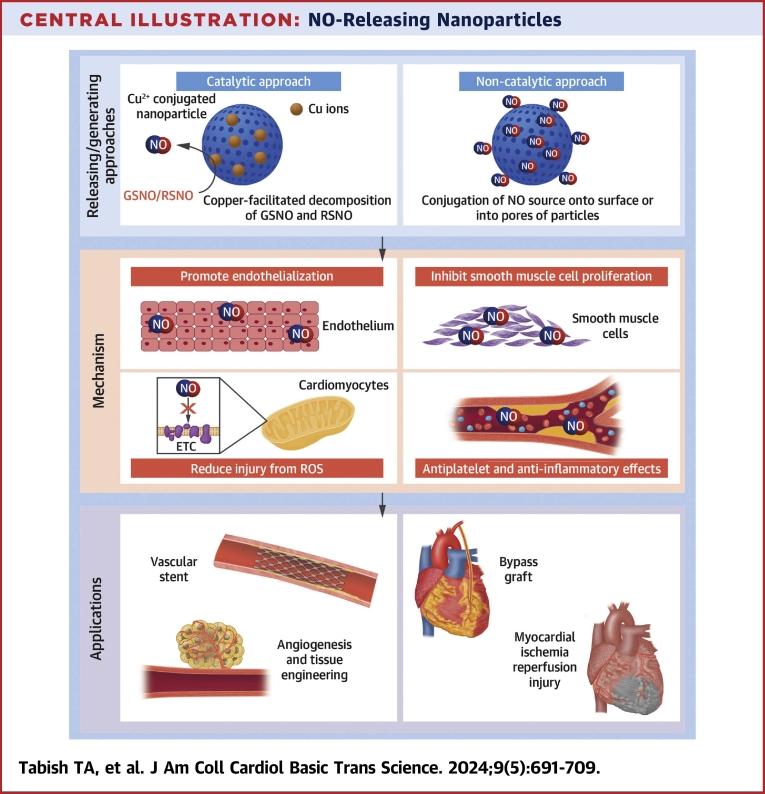
NO-Releasing Nanoparticles Focus on nitric oxide releasing and generating nanomaterials and overview of their mechanism of action for treating cardiovascular diseases.

For example, there are many variables in NP design that require optimization for any given application, eg, nanomaterial selection, structural design, physicochemical properties, and fabrication procedures. There are multiple choices for the source of NO and how these are incorporated into the NPs. Any formulation must be stable during storage and provide targeted, reproducible NO generation with clearly defined kinetics and duration. It is a major limitation that we lack methods for real-time monitoring/tracking of NO release in vivo, where there are also challenges with systemic administration and concerns about long-term toxicity. It is therefore important to understand issues relating to cellular accumulation, breakdown, and elimination of NPs. Large animal work is needed to generate more meaningful and translatable findings, particularly in relation to the development of stents, but also for I/R therapies.

One major barrier to progress with NO-releasing nanoformulations is the need for multidisciplinary teams spanning materials science, engineering, chemistry, biotechnology, pharmacology, and cardiovascular medicine. It is hoped that interdisciplinary fellowships will help bridge that gap. Nevertheless, we believe that recent advances in nanotechnology, particularly in relation to materials and fabrication techniques, will boost research into NO-releasing/generating formulations. For example, since the discovery of graphene in 2003, it has been widely investigated for image-guided drug delivery and sensing applications mainly for cancer and infectious diseases. However, graphene-based nanomaterials have not yet been developed for NO storage and release purposes despite highly favorable characteristics, eg, ultra-small size, exceptionally high surface area, and a wide variety of shapes and morphologies (graphene quantum dots, graphene oxide, reduced graphene oxide, porous graphene nanosheets, graphene nanoplatelets, graphene aerogels/hydrogels, graphene foam).

NO-releasing NPs could also be extended to other CVD applications, such as angina and peripheral vascular disease. Modification of NPs may help minimize toxicity and adverse effects, eg, by favoring specific metabolic pathways or preventing drugs crossing the blood-brain barrier. It is also possible to adapt NPs as a delivery vehicle for other payloads such as hydrogen sulfide, carbon monoxide, nucleic acids, siRNA, DNA, peptides, proteins, and antibodies. Clearly this field is still in its infancy, but the detailed study of NPs for cardiovascular disease applications is an attractive prospect that promises to open up a new paradigm of highly tunable drug delivery.

## Funding Support and Author Disclosures

This work was supported by a British Heart Foundation (BHF) Fellowship (FS/ATA/21/20015) to Dr Tabish. Work in the authors’ laboratory is funded by BHF programme grant (RG/18/12/34040) to Dr Lygate. All other authors have reported that they have no relationships relevant to the contents of this paper to disclose.
